# Livestock ownership and microbial contamination of drinking-water: Evidence from nationally representative household surveys in Ghana, Nepal and Bangladesh

**DOI:** 10.1016/j.ijheh.2017.09.014

**Published:** 2018-01

**Authors:** Nicola A. Wardrop, Allan G. Hill, Mawuli Dzodzomenyo, Genevieve Aryeetey, Jim A. Wright

**Affiliations:** a Geography and Environment, University of Southampton, Southampton, Hampshire, UK; b Social Sciences, University of Southampton, Southampton, Hampshire, UK; c Ghana School of Public Health, University of Ghana, Legon, Accra, Ghana

**Keywords:** Drinking-water, Microbial contamination, Livestock, Zoonoses, Developing countries, *Escherichia coli*

## Abstract

**Background:**

Current priorities for diarrhoeal disease prevention include use of sanitation and safe water. There have been few attempts to quantify the importance of animal faeces in drinking-water contamination, despite the presence of potentially water-borne zoonotic pathogens in animal faeces.

**Objectives:**

This study aimed to quantify the relationship between livestock ownership and point-of-consumption drinking-water contamination.

**Methods:**

Data from nationally representative household surveys in Nepal, Bangladesh, and Ghana, each with associated water quality assessments, were used. Multinomial regression adjusting for confounders was applied to assess the relationship between livestock ownership and the level of drinking-water contamination with *E. coli*.

**Results:**

Ownership of five or more large livestock (e.g. cattle) was significantly associated with drinking-water contamination in Ghana (RRR = 7.9, 95% CI = 1.6 to 38.9 for medium levels of contamination with 1–31cfu/100 ml; RRR = 5.2, 95% CI = 1.1–24.5 for high levels of contamination with >31cfu/100 ml) and Bangladesh (RRR = 2.4, 95% CI = 1.3–4.5 for medium levels of contamination; non-significant for high levels of contamination). Ownership of eight or more poultry (chickens, guinea fowl, ducks or turkeys) was associated with drinking-water contamination in Bangladesh (RRR = 1.5, 95% CI = 1.1–2.0 for medium levels of contamination, non-significant for high levels of contamination).

**Conclusions:**

These results suggest that livestock ownership is a significant risk factor for the contamination of drinking-water at the point of consumption. This indicates that addressing human sanitation without consideration of faecal contamination from livestock sources will not be sufficient to prevent drinking-water contamination.

## Highlights

• Owning livestock can increase the risk of drinking-water contamination.
• Large livestock were associated with water contamination in Ghana and Bangladesh.
• Poultry were associated with drinking-water contamination in Bangladesh.
• Livestock were not significantly correlated with water contamination in Nepal.
• Livestock excreta should be considered for the prevention of water contamination.

## 1 Introduction

Building on the Millennium Development Goals, the Sustainable Development Goals (SDGs), which were adopted in September 2015, include a focus on safe water and sanitation (United Nations General Assembly, 2015). SDG 6 aims to “ensure availability and sustainable management of water and sanitation for all”, with proposed monitoring of the percentage of the population using safely managed drinking-water services (Sustainable Development Solutions Network, 2015). These are defined as a basic drinking-water source (piped water; boreholes or tubewells; protected dug wells; protected springs and rainwater) which is located on household premises and available when needed. Such services should also be free of faecal and priority chemical (identified nationally, but globally fluoride and arsenic) contamination and/or regulated by a competent authority (World Health Organization, 2015). The use of contaminated drinking-water (along with inadequate sanitation and hygiene) is a key contributing factor in diarrhoeal disease, particularly in low and middle-income settings (Prüss-Ustün et al., 2014). Current priorities with regard to sanitation focus primarily on the management of human faecal matter, and largely ignore the management of faecal matter from domestic animals, despite their contribution, as a group, of 85% of global animal faecal waste (Dufour et al., 2012).

In 2015, an estimated 844 million people globally were not using a basic source of drinking-water, and 159 million people were relying on untreated surface water, which is highly susceptible to contamination (World Health Organization and UNICEF, 2017). While 89% of the global population were using improved water sources (defined as those specifically designed to avoid contamination from outside) in 2012, water from these sources is also frequently found to be contaminated, or to become contaminated during storage within the home, with a recent systematic review indicating that many improved sources contained faecal indicator bacteria at levels above World Health Organization guideline values (Bain et al., 2014). Globally, the population using unimproved water sources, or improved water sources with faecal contamination in 2012 was estimated to be 1.9 billion (Bain et al., 2014).

Overall, diarrhoeal disease accounted for 1.4 million deaths in 2010, including 17.4% of deaths in children aged 28 days to 1 year, and 11.9% of deaths in children aged 1–4 years. Diarrhoeal disease is the 4th leading cause for years of life lost globally (Lozano et al., 2012). Of these deaths, 502,000 have been attributed to inadequate drinking-water and 280,000 to inadequate sanitation (Prüss-Ustün et al., 2014). In addition to diarrhoeal disease, inadequate drinking-water, sanitation and hygiene has complex impacts on undernutrition, growth stunting and environmental enteropathy, with subsequent life-long consequences, although these effects are difficult to quantify due to a lack of data (Clasen et al., 2014).

Microbial testing for faecal indicator bacteria (e.g. thermotolerant coliforms or *Escherichia coli*) is recommended to detect faecal contamination. The indicator bacteria groups used to detect faecal contamination are common to humans, livestock and many wildlife species. Thus, detection of faecal indicator bacteria may indicate contamination by human faeces, animal faeces or both (Mackay and Oxford, 1954, Meays et al., 2004). There are a number of potentially water-borne zoonotic pathogens which can be detrimental to human health, such as *Salmonella* spp*, E. coli* 0157:H7, *Campylobacter* and *Cryptosporidium parvum* (Cotruvo et al., 2004, Dufour et al., 2012). Several studies have detected significant positive correlations between domestic livestock and poultry contact and diarrhoeal disease in humans (Conan et al., 2017, El-Tras et al., 2015, Kaur et al., 2017, Zambrano et al., 2014). However, there have been few attempts to quantify the importance of animal faeces in the contamination of drinking-water. Those studies which have been conducted indicate that domestic animals contribute significant levels of faecal contamination to water sources (Daniels et al., 2015, Schriewer et al., 2015). The risks to human health posed by animal faeces are not well understood, however, and it is often assumed that due to the species-specific nature of many pathogens, contamination by animal faeces presents less risk than that by human faeces (Dufour et al., 2012). A better understanding of human versus animal sourced water contamination patterns, and the subsequent implications for human health, is required to enable effective and efficient interventions.

The present study aims to assess the potential influence of ownership of livestock, including poultry, on drinking-water contamination. We used three unique, nationally representative household surveys with associated water quality modules from Ghana, Bangladesh and Nepal, to address this aim. When analysis was undertaken, these were the only countries available with published micro-data from household surveys that included a water quality module. The statistical correlation between ownership of domestic animals and the presence of faecal indicator bacteria in water samples at the point of consumption was assessed, after controlling for confounding factors.

## 2 Materials and methods

### 2.1 Data

Data from the Ghana Living Standards Survey Round 6 (GLSS 6), Bangladesh Multiple Indicator Cluster Survey 2012–2013 (MICS) and Nepal MICS 2014 were used. These were nationally representative household surveys, which recorded household characteristics including housing conditions and household agriculture, and were conducted from October 2012 to October 2013 (Ghana), December 2012 to April 2013 (Bangladesh) and January to June 2014 (Nepal). In terms of the seasons, in Ghana the survey was conducted over a full calendar year (in each region); and in Bangladesh and Nepal the surveys were conducted mainly during the dry season. Each of these studies used a two-stage cluster sampling design to provide estimates representative at the national, urban versus rural, and sub-national levels (region in Ghana, district in Bangladesh and ecological zone in Nepal). The data collection procedures were approved by the relevant institutional review board in each country.

Nested within these surveys were additional water quality modules, for which households were selected randomly from within each cluster (three households per cluster in Ghana, one per cluster in Bangladesh, and three per cluster in Nepal). Respondents were requested for “a glass of water which you would give a child to drink”. The water provided was tested for *E. coli* as an indicator of faecal contamination by incubating 100 ml of the sample on Compact Dry EC growth media plates (Nissui, Japan), after filtration through a 0.45 μm filter (Millipore Microfil). Incubation was carried out at ambient temperature for 24 h, after which *E. coli* colonies were enumerated and recorded as colony forming units (CFUs) per 100 ml of water. The water quality testing procedures used were the same in all three surveys. Out of the overall sample sizes of 16,772 households (Ghana); 55,120 households (Bangladesh), and 12,975 households (Nepal), 2972; 2592; and 1492 had associated information on water contamination at the point of consumption, respectively.

As identified in Wright et al. (2016), CFU counts in these datasets exhibited preferential recording of values ending in zero. Since ‘heaped’ values such as 10 and 100 fall at class intervals, this makes analysis using the WHO risk categories of 1–10, 11–100, and 100–1000 problematic (World Health Organization, 1997). Thus, *E. coli* CFU data were categorised into three groups representing no contamination (0 CFU/100 ml); medium contamination (1–31 CFU/100 ml) and high contamination (>31 CFU/100 ml). These cut-offs were selected by pooling the data from the three countries, excluding observations with 0 CFU/100 ml, then selecting the median value to ensure approximately equal counts within each category and comparability of analyses between countries. Livestock ownership was the primary factor of interest: the surveys included information on ownership (and numbers owned for each livestock species) of draught animals (e.g. donkeys, horses or bullocks), cattle, yak, buffalo, sheep, goats, pigs, and poultry (not all countries included all livestock types). Aggregate livestock categories were created by summing the number of animals owned in groups of livestock species: (1) large livestock (cattle, draught animals, yak and buffalo); (2) small livestock (sheep, goats and pigs); and (3) poultry (chickens, guinea fowl, ducks and turkeys were aggregated where these were recorded separately).

Variables relating to several important confounding factors for the assessment of faecal contamination of water were created. The level of faecal contamination in water from different source types can vary widely, including between different improved water sources (Bain et al., 2014, Shields et al., 2015). Thus, the source of drinking-water was defined in the following categories: (1) piped to premises; (2) standpipe, tanker or neighbours tap; (3) tubewell or borehole; (4) protected well or spring; (5) unprotected well or spring; (6) surface water; (7) sachet or bottled water; or (8) rain water collection. To avoid very small cell counts for Bangladesh, categories (3) and (4) were merged. Lack of improved sanitation is a key risk factor for environmental contamination with human faeces and, thus, contamination of drinking-water. Therefore, access to improved sanitation (including water closet, pit latrine, Kumasi ventilated improved pit latrine (KVIP), and public toilet) was categorised for each country. Water which is stored within the home has been found to be at higher risk of faecal contamination than water taken directly from the source, most likely due to post-collection contamination (Shields et al., 2015, Wright et al., 2004). To address this, we created a variable categorizing water as not stored, stored in a covered vessel or filter, or stored in an uncovered vessel. Access to safe drinking-water has also been shown to vary by socio-economic status (Yang et al., 2013). For Ghana, regionally deflated total expenditure (GH₵) per day, per adult equivalent was used as a proxy for household socio-economic status and for Bangladesh and Nepal, wealth quintile was used.

### 2.2 Statistical analysis

The percentage of point-of-consumption water samples that fell within each contamination category was calculated by ownership status of livestock groups (large livestock; small livestock; poultry). The mean and median number of each livestock species (or aggregate group) owned per household were calculated to summarise their relative abundance, excluding households where the number owned was zero. In addition, the percentage of point-of-consumption water samples within each contamination category was calculated for each of the categorical confounder variables: water source, access to improved sanitation and water storage. For further analysis, the aggregate livestock classes were categorised as follows due to over-dispersion: large livestock (0; 1–4; 5 or more), small livestock (0; 1–10; 11 or more), and poultry (0; 1–20; 21 or more). These cut-offs were selected to avoid small cell counts and to ensure comparability between countries.

Since there was evidence that the proportional odds assumption required for ordinal regression was violated, the relationship between household ownership of livestock and the level of point-of-consumption water contamination was assessed using multinomial logistic regression analysis. Unadjusted and adjusted (accounting for the confounding factors water source, improved sanitation, water storage and expenditure/wealth quintile by including these variables in a multivariable regression) relative risk ratios (RRRs) were calculated for binary aggregate livestock variables (ownership versus non-ownership) and categorised aggregate livestock variables (categorised numbers of animals owned in each group). The GLSS6 additionally included data on household spending on livestock feed and agricultural land ownership by household members. Since use of animal feed suggested that animals were not free-ranging and land ownership suggested livestock might be kept away from the home, we therefore also included these terms in our analysis for Ghana. All statistical analyses were carried out for all three study countries in Stata 13.1 (StataCorp LP, Texas) using the *svy* commands to account for survey design (this command accounts for characteristics of complex survey designs such as sampling weights, clustered sampling, and stratification).

## 3 Results

Rates of livestock ownership varied by type and country (see Table 1). Large livestock ownership was considerably more common in Bangladesh and Nepal (35.4% and 54.1% respectively) compared to Ghana (4.3%); small livestock ownership was more common in Nepal (48.8%) compared to Ghana and Bangladesh (21.3% and 19.3% respectively); and poultry ownership was more common in Bangladesh (55.2%) compared to Ghana and Nepal (28.3% and 35.1% respectively). Herd or flock sizes varied between livestock type and country (see Table 2): despite lower ownership rates in Ghana, mean and median herd/flock sizes were larger for all livestock types. Mean herd/flock sizes were largest for poultry in all three study countries: 19.2 in Ghana, 6.7 in Bangladesh and 7.2 in Nepal. Due to the high mean flock size and high ownership rate compared to other livestock types, poultry are the most numerous livestock in both Ghana and Bangladesh.

**Table 1:** Percentage of point-of-consumption water samples in each contamination category, by livestock ownership status.

Livestock type	Owner	Ghana (N = 2972)				Bangladesh (N = 2592)				Nepal (N = 1492)			
		<1 CFU/100 ml	1–31 CFU/100 ml	>31 CFU/100 ml	Overall	<1 CFU/100 ml	1–31 CFU/100 ml	>31 CFU/100 ml	Overall	<1 CFU/100 ml	1–31 CFU/100 ml	>31 CFU/100 ml	Overall
Large	Yes	6.5%	34.1%	59.4%	**4.3%**	37.5%	40.2%	22.3%	**35.4%**	12.5%	41.8%	45.8%	**54.1%**
	No	29.0%	28.5%	42.6%	**95.7%**	37.2%	34.8%	28.0%	**64.6%**	24.2%	43.5%	32.3%	**45.9%**
Small	Yes	14.1%	27.8%	58.1%	**21.3%**	35.6%	42.0%	22.5%	**19.3%**	13.6%	42.5%	43.9%	**48.8%**
	No	31.7%	29.0%	39.3%	**78.7%**	37.7%	35.5%	26.8%	**80.7%**	21.9%	42.7%	35.5%	**51.2%**
Poultry	Yes	13.7%	24.1%	62.3%	**28.3%**	36.8%	39.9%	23.3%	**55.2%**	14.6%	38.7%	46.7%	**35.1%**
	No	33.6%	30.6%	35.8%	**71.7%**	37.9%	32.8%	29.4%	**44.8%**	19.6%	44.7%	35.7%	**64.9%**
***Overall***		***28.0%***	***28.7%***	***43.3%***		***37.3%***	***36.7%***	***26.0%***		***17.8%***	***42.6%***	***39.6%***

**Table 2:** Livestock herd/flock size summary data, excluding non-owners for each species.

Livestock species	Ghana		Bangladesh		Nepal	
	Mean	Median	Mean	Median	Mean	Median
Draught/horses	2.8	2	a	a	2.3	2
Cattle	8.9	5	2.3	2	2.9	2
Yak	NA	NA	NA	NA	22.1	16
Buffalo	NA	NA	NA	NA	1.7	2
*Large livestock combined*	***8.9***	***5***	***2.3***	***2***	***3.5***	***3***
Sheep	14.7	5	3.4	3	11.2^b^	2.5^b^
Goats	6.7	5	2.4	2	5.0	3
Pigs	5.9	4	2.1	2	1.8	1
*Small livestock combined*	***13.7***	***7***	***2.5***	***2***	***5.4***^b^	***3***^b^
Chickens	16.0	10	NA	NA	NA	NA
Guinea Fowl	13.6	10	NA	NA	NA	NA
Duck	5.5	4	NA	NA	NA	NA
Turkey	5.8	4	NA	NA	NA	NA
*All poultry combined*^c^	***19.2***	***13***	***6.7***^b^	***5***^b^	***7.2***	***5***

a Only one household owned draught animals in Bangladesh, hence summary statistics are not provided.

b Note recording of animal numbers was truncated at 95, with one or more observations reporting >95 animals in the categories marked (these observations were treated as equal to 95 animals).

c Poultry were reported in an aggregate category for Bangladesh and Nepal.

The percentage of water samples which were classified as highly contaminated (>31 CFU/100 ml) was higher for households that owned livestock than for households that did not own livestock in Ghana and Nepal, but not Bangladesh (see Table 1). This was consistent across all livestock species. Patterns of water contamination (regardless of livestock ownership) also varied between countries. The highest proportion of uncontaminated water samples was found in Bangladesh (37.3%), with lower proportions for the medium (36.7%) and high (26.0%) contamination categories. The highest proportion of highly contaminated water samples was detected in Ghana (43.3%), with lower proportions in the medium (28.7%) and no (28.0%) contamination categories. In Nepal, the greatest proportion of samples were within the medium contamination category (42.6%), and the lowest proportion in the no contamination category (17.8%).

Assessment of contamination levels broken down by water source, access to improved sanitation and water storage (see Table 3) highlights important differences. The percentage of point-of-consumption water samples which were highly contaminated was substantially lower for sachet or bottled water than other water source types in Ghana (3.5% compared to 20.5% in water piped to premises, the next lowest water source type), but only marginally lower in Nepal (30.5% compared with 33.3%; this water source was not defined in the Bangladesh data). For Ghana and Nepal, the highest proportion of samples that were highly contaminated came from surface water sources (86.6%, and 65.2% respectively), while for Bangladesh the highest proportion of highly contaminated water samples were piped to premises (60.3%). Those with improved sanitation had lower proportions of highly contaminated water samples than those without in all three countries, although the difference was reasonably modest for Bangladesh and Nepal. Stored water was more likely to be contaminated, although again this difference was most marked for Ghana (Table 3).

**Table 3:** Percentage of point-of-consumption water samples in each contamination category, by water source, sanitation type and water storage status.

Variable	Category	Ghana			Bangladesh			Nepal		
		0 CFU	1–31 CFU	>31 CFU	0 CFU	1–31 CFU	>31 CFU	0 CFU	1–31 CFU	>31 CFU
Water source	Piped to premises	34.5%	45.1%	20.5%	22.8%	16.8%	60.3%	27.2%	39.6%	33.3%
	Standpipe, tanker or neighbours tap	13.8%	29.1%	57.1%	44.5%	25.2%	30.3%	11.4%	44.5%	44.1%
	Tubewell or borehole*	10.5%	29.2%	60.3%	39.2%	38.6%	22.2%	18.5%	43.9%	37.6%
	Protected well or spring*	11.8%	27.5%	60.7%				9.6%	44.5%	45.9%
	Unprotected well or spring	12.2%	25.1%	62.6%	23.7%	28.3%	48.0%	3.8%	39.8%	56.4%
	Surface water	5.1%	8.4%	86.6%	4.6%	52.5%	42.9%	9.1%	25.7%	65.2%
	Sachet or bottled water	68.8%	27.7%	3.5%				34.0%	35.5%	30.5%
	Rainwater	7.1%	42.3%	50.6%						
Sanitation	Improved	31.5%	29.1%	39.4%	38.6%	36.3%	25.2%	19.7%	41.8%	38.5%
	Unimproved	13.6%	26.9%	59.5%	33.3%	38.3%	28.4%	12.6%	44.9%	42.6%
Water storage	Not stored	47.1%	28.6%	24.2%	49.8%	31.8%	18.5%	17.6%	44.6%	37.9%
	Stored in covered vessel or filter	17.4%	29.7%	52.9%	32.9%	35.9%	31.3%	27.6%	40.5%	31.9%
	Stored in uncovered vessel	6.4%	20.1%	73.5%	35.6%	41.2%	23.2%	5.1%	42.1%	52.7%

For Bangladesh, the water sources “tubewell or borehole” and “protected well or spring” were merged due to a small number of households reporting the latter water source.

Unadjusted and adjusted RRRs associated with livestock ownership (large livestock, small livestock, and poultry) are presented in Table 4, Table 5, Table 6 for Ghana, Bangladesh and Nepal respectively. The unadjusted RRRs indicate that ownership of livestock is significantly associated with increased risk of both medium and high contamination of drinking-water at the point of consumption in Ghana and Nepal (p < 0.05). In Bangladesh, significant relationships are seen for large livestock and poultry (but not small livestock), for the medium contamination group only. After accounting for the effects of important confounders (water source, access to improved sanitation, water storage practices and expenditure/wealth quintile), large livestock ownership as a binary variable was significantly associated with medium levels of contamination of drinking-water at the point of consumption in Ghana only (adjusted RRR [aRRR] = 4.1, p = 0.05, see Table 4). In Ghana, ownership of five or more large livestock was significantly associated with medium (aRRR = 7.9, p = 0.01) or high (aRRR = 5.2, p = 0.04) contamination levels in drinking-water at the point of consumption (Table 4). In Bangladesh, ownership of five or more large livestock was significantly associated with medium contamination levels (aRRR = 2.4, p = 0.004), but not high contamination levels. Furthermore, ownership of eight or more poultry was also associated with medium contamination levels (aRRR = 1.5, p = 0.01), but not high contamination levels in Bangladesh. In Nepal, there was no evidence for a relationship between livestock ownership and drinking-water contamination while adjusting for confounding factors (p > 0.5 for all adjusted analyses). In general, model results also indicated elevated RRR in households which: were in the lowest wealth quintiles; used surface water or unprotected well/spring sources; lacked improved sanitation (although this was not statistically significant); stored their water in an uncovered container before use (for Ghana and Nepal); or stored their water in an uncovered or covered container or filter before use (for Bangladesh). Model coefficients and statistical significance varied between countries and the specific model being fit. When additional coefficients for household spending on animal feed and household agricultural land ownership were included in the models for Ghana, these were not significant and did not significantly interact with livestock ownership.

**Table 4:** Unadjusted and adjusted RRRs related to ownership (versus non-ownership) of large livestock, small livestock and poultry, and categorised numbers of animals owned within each group, for medium and high contamination categories in comparison to the no contamination category in Ghana. N = 2972 (unadjusted) and N = 2822 (adjusted).

Livestock type	Ownership status/number owned	1–31 CFU		>31 CFU		1–31 CFU		>31 CFU	
		Unadjusted RRR (95% CI)	p-value	Unadjusted RRR (95% CI)	p-value	Adjusted RRR^a^ (95% CI)	p-value	Adjusted RRR^a^ (95% CI)	p-value
Large	Non-owner	Ref		Ref		Ref		Ref	
	Owner	5.3 (2.4–11.5)	<0.001*	6.2 (3.2–11.8)	<0.001*	4.1 (1.0–16.5)	0.05*	3.1 (0.9–11.4)	0.08
Small	Non-owner	Ref		Ref		Ref		Ref	
	Owner	2.1 (1.5–3.0)	<0.001*	3.3 (2.4–4.7)	<0.001*	1.00 (0.7–1.5)	0.99	0.85 (0.6–1.3)	0.43
Poultry	Non-owner	Ref		Ref		Ref		Ref	
	Owner	1.9 (1.4–2.6)	<0.001*	4.3 (3.2–5.7)	<0.001*	0.98 (0.7–1.4)	0.89	1.2 (0.8–1.7)	0.34
Large	0	Ref		Ref		Ref		Ref	
	1–4	3.5 (1.3–9.5)	0.02*	6.4 (2.5–16.4)	<0.001*	1.3 (0.4–4.1)	0.61	1.3 (0.5–3.8)	0.60
	5+	7.1 (2.6–19.6)	<0.001*	5.9 (2.6–13.6)	<0.001*	7.9 (1.6–38.9)	0.01*	5.2 (1.1–24.5)	0.04*
Small	0	Ref		Ref		Ref		Ref	
	1–4	2.9 (1.7–5.0)	<0.001*	3.8 (2.4–6.0)	<0.001*	1.3 (0.7–2.3)	0.46	0.9 (0.6–1.4)	0.61
	5+	1.9 (1.2–2.8)	0.002*	3.1 (2.1–4.7)	<0.001*	0.9 (0.5–1.5)	0.59	0.9 (0.5–1.3)	0.48
Poultry	0	Ref		Ref		Ref		Ref	
	1–7	1.8 (1.1–2.8)	0.01*	3.4 (2.3–5.0)	<0.001*	0.8 (0.5–1.5)	0.63	0.9 (0.6–1.5)	0.77
	8+	2.0 (1.4–2.9)	<0.001*	4.7 (3.3–6.7)	<0.001*	1.0 (0.7–1.5)	0.91	1.3 (0.9–2.0)	0.17

Ref = reference category. *Statistically significant (p ≤ 0.05). ^a^Adjusted for water source, presence of improved sanitation, water storage status and expenditure, but not other livestock ownership categories.

**Table 5:** Unadjusted and adjusted RRRs related to ownership (versus non-ownership) of large livestock, small livestock and poultry, and categorised numbers of animals owned within each group, for medium and high contamination categories in comparison to the no contamination category in Bangladesh. N = 2592 (unadjusted) and N = 2541 (adjusted).

Livestock type	Ownership status/number owned	1–31 CFU		>31 CFU		1–31 CFU		>31 CFU	
		Unadjusted RRR (95% CI)	p-value	Unadjusted RRR (95% CI)	p-value	Adjusted RRR^a^ (95% CI)	p-value	Adjusted RRR^a^ (95% CI)	p-value
Large	Non-owner	Ref		Ref		Ref		Ref	
	Owner	1.1 (0.9–1.4)	0.24	0.8 (0.6–1.0)	0.10	1.1 (0.9–1.4)	0.26	1.0 (0.8–1.3)	0.92
Small	Non-owner	Ref		Ref		Ref		Ref	
	Owner	1.3 (0.8–1.6)	0.08	0.9 (0.7–1.2)	0.44	1.2 (1.0–1.6)	0.11	1.1 (0.8–1.5)	0.63
Poultry	Non-owner	Ref		Ref		Ref		Ref	
	Owner	1.3 (1.0–1.6)	0.05*	0.8 (0.6–1.1)	0.15	1.2 (0.7–1.5)	0.10	1.1 (0.9–1.5)	0.35
Large	0	Ref		Ref		Ref		Ref	
	1–4	1.1 (0.9–1.4)	0.47	0.8 (0.6–1.0)	0.08	1.1 (0.9–1.4)	0.52	1.0 (0.7–1.3)	0.96
	5+	2.3 (1.3–4.2)	0.007*	1.1 (0.4–2.6)	0.89	2.4 (1.3–4.5)	0.004*	1.5 (0.6–3.8)	0.40
Small	0	Ref		Ref		Ref		Ref	
	1–4	1.2 (0.9–1.6)	0.14	0.8 (0.6–1.2)	0.30	1.2 (0.9–1.6)	0.18	1.0 (0.7–1.4)	0.83
	5+	1.5 (0.8–2.8)	0.26	1.2 (0.5–2.9)	0.73	1.5 (0.8–2.9)	0.25	1.4 (0.5–3.6)	0.52
Poultry	0	Ref		Ref		Ref		Ref	
	1–7	1.2 (0.9–1.5)	0.21	0.8 (0.6–1.1)	0.25	1.1 (0.9–1.4)	0.43	1.2 (0.9–1.6)	0.28
	8+	1.5 (1.1–2.0)	0.01*	0.8 (0.5–1.1)	0.14	1.5 (1.1–2.1)	0.01*	1.0 (0.7–1.5)	0.93

Ref = reference category. *Statistically significant (p ≤ 0.05). ^a^Adjusted for water source, presence of improved sanitation, water storage status and wealth quintile, but not other livestock ownership categories.

## 4 Discussion

Increasing access to safe water is a key component in the recently adopted Sustainable Development Goals, aiming to prevent water-borne diseases and exposure to chemical contaminants. The prevention of contamination of water supplies by human faecal material, for example via the provision of improved sanitation, plays a major part in progress towards this goal. However, exposure to animal faecal material can also pose a risk to human health, although there is limited evidence regarding the impact of livestock populations on faecal contamination of drinking-water. Here, we provide evidence that livestock ownership (in particular large livestock animals and poultry) is a significant risk factor for the contamination of drinking-water at the point of consumption (i.e. in “a glass of water which you would give a child to drink”) in Ghana and Bangladesh, but not Nepal. This has important implications for future work aiming to improve access to safe drinking-water.

Unadjusted regression results indicate that ownership of all types of livestock (large livestock, small livestock and poultry) was significantly associated with an increased risk of contamination of drinking-water at the point of consumption in Ghana and Nepal. In Bangladesh, large livestock and poultry ownership were significantly associated with drinking-water contamination, but not small livestock. After adjustment for water source type, access to improved sanitation, water storage practices and expenditure/wealth quintile (as a proxy for socio-economic status) the regression results were substantially different. In Nepal, none of the livestock ownership variables (binary or categorical) were significantly associated with contamination of drinking-water after adjusting for confounding factors. However, in Ghana, a significant association was observed for large livestock as both a binary ownership variable and for households owning five or more large livestock, and in Bangladesh categorical variables for large livestock (five or more) and poultry (eight or more) ownership were significantly associated with water contamination.

These results suggest that livestock populations contribute to the faecal contamination of drinking-water in Ghana and Bangladesh, but not Nepal. In all three of the study countries, the main agricultural system is smallholder farming, mainly sedentary mixed crop-livestock systems, although in some areas (e.g. mountainous parts of Nepal where yak farming is common), livestock keeping can be migratory. In poorer regions, livestock ownership is also used as a means of financial security (Ministry of Food and Agriculture, 2010). The use of extensive grazing and free-ranging of animals, which is common in each of the study countries, can enable widespread contamination of the environment, including around homes, with faecal matter. There are differences in livestock herd composition and size between countries: for example, livestock ownership is most common in Nepal, and cattle herd sizes are largest in Ghana. Medium and high contamination levels were associated with greater large-stock herd sizes in Bangladesh and Ghana (Table 4, Table 5), yet such larger herds might be expected to be kept outside household compounds. Similarly, lack of spending on animal feed and non-ownership of land, both potential indicators of free-ranging livestock husbandry within the household compound, were not associated with water contamination in Ghana. The non-significance of livestock ownership in Nepal may be due to differing livestock husbandry practices: for example, the largest herd sizes in Nepal consisted of Yak, which tend to live at high altitudes and are migratory, which may result in lower potential for faecal contamination of household drinking-water by Yak faeces. However, there may be further inter-country differences which have not been possible to assess using the available data. Differences in livestock husbandry practices (e.g. whether livestock are generally allowed to enter domestic environments), climatic factors (e.g. rainfall patterns may influence the likelihood of contamination of drinking water sources from livestock sources), or household behaviors (e.g. ensuring animal faeces is disposed of from the domestic environment) may also play a role.

Considerable attention has been paid to the contamination of drinking-water supplies, and the contribution of drinking-water quality, sanitation and hygiene to diarrhoeal disease. However, there has been less attention to the influence of livestock ownership on water contamination and risk of diarrhoeal disease. A recent systematic review indicated that 69% of studies assessing the relationship between domestic animal husbandry and diarrhoeal disease in humans reported a significant positive association, and this increased to 95% when considering only studies assessing specific diarrhoeal pathogens (i.e. excluding studies looking at non-pathogen specific diarrhoea) (Zambrano et al., 2014). Several studies have detected positive associations between exposure to poultry and *Campylobacter* spp. infections (Grados et al., 1988, Lengerh et al., 2013), with one also noting a significantly higher odds of *Campylobacter* spp. infections in homes without running water (Georges-Courbot et al., 1990). Similarly, *Giardia* spp. infections have been found to be positively associated with contact with ruminants (Wegayehu et al., 2013), poultry (El-Tras et al., 2015, Mahmud et al., 1995) and a combination of these livestock types (Coles et al., 2009). In Bangladesh, presence of livestock faeces in household compounds was associated with *E. coli* contamination of soil and presence of chicken faeces in particular was associated with both soil and stored water contamination with *E. coli* (Ercumen et al., 2017). The contamination of drinking-water supplies by livestock in India has recently been demonstrated by Schriewer et al. (2015). Microbial source tracking methods indicated that animal associated faecal matter was present in 4.7% of public and 15% of private tube wells, compared to 4.7% of public and 2.4% of private tube wells for human faecal matter. Considering stored water within the home, 20% of stored water samples were contaminated with faecal matter from human sources and 52% with faecal waste from animal sources. These results indicated that (within the specific study area in India) livestock were the most common source of faecal contamination in drinking-water at source, and in stored water within the home, with increased levels of contamination in stored water compared to water at source (Schriewer et al., 2015).

In the context of previous evidence demonstrating faecal contamination of drinking-water and the wider domestic environment by livestock, and increased risk of drinking-water contamination when water is stored within the home (Shields et al., 2015, Wright et al., 2004), it is likely that livestock contribute to the faecal contamination of drinking-water both at source and during storage within the home. In many resource poor settings, human populations live in close contact with livestock, providing opportunity for the contamination of the household environment with animal faecal matter. It is important to recognise that faecal matter contaminating drinking-water supplies does not necessarily arise solely from human sources, and contamination can occur both at source and during storage within the home. Thus, interventions to improve human sanitation or provide improved water sources will not necessarily be sufficient to prevent the consumption of faecally contaminated water: a broader approach may be necessary. This could include, for example, the promotion of specific husbandry methods, hand-washing practices (Conan et al., 2017) or water treatment within the home, although further evidence of the effectiveness of these interventions would be required.

Domestic livestock play a vital role in rural livelihoods, particularly in resource-poor settings, contributing to nutrition, financial savings and income, the production of food crops via draught power and manure, and the provision of (dung-based) fuel (Randolph et al., 2007). Thus, livestock ownership can have significant health benefits due to improved nutrition and higher socio-economic status. For example, a recent study found a reduced prevalence of child stunting in households which owned more livestock in Ethiopia and Uganda, although this association did not extend to Kenya (Mosites et al., 2015). Kaur et al. (2017) also identified an overall protective effect of livestock ownership against child stunting, using pooled data from 30 African countries. However, the close proximity between human and domestic livestock populations in many low-income settings substantially increases the risk of zoonotic diseases, including some which may be water-borne (Cotruvo et al., 2004, Molyneux et al., 2011). The pooled analysis of 30 African countries found no overall effect of livestock ownership on child diarrhoea but did detect an overall detrimental effect on child mortality, although it is important to note that there was substantial heterogeneity in the relationships observed (including in the direction of the relationship) for each outcome variable between countries (Kaur et al., 2017). Further research is needed to quantify both the positive and negative impacts of livestock ownership, enabling the development of appropriate interventions to maximise net benefits while minimising potential health risks, such as contamination of drinking-water and the transmission of water-borne zoonoses.

It is important to bear in mind the limitations of this study. The use of water at the point of consumption prevents the differentiation between contamination at source or during storage within the home. The water quality module of these surveys also conducted sampling of water samples taken directly from the source (from a reduced number of households). However, detection of associations between household livestock ownership and faecal contamination of water at source is complicated by several factors, including the distance between a homestead and the water source and livestock ownership patterns in other households close to the water source. Thus, we did not assess contamination of water from source. Based on the data available, it was not possible to identify the physical location of livestock in relation to the household. It may be anticipated that larger herds or flocks would be housed at a greater distance from the household and therefore would be less likely to contribute to the contamination of water supplies, and as previously noted, some of the animal herds may be migratory. Further analysis of animal husbandry was not possible due to the different focus of the household survey used, but in future, efforts should be made to address the influence of animal husbandry practices on the contamination of drinking-water. The administration of more specific surveys would enable the inclusion of additional information, such as proximity of livestock to water sources and households. Given the variation in livestock ownership by rurality and region, these variables may also have been included in the regression model. However, these variables were collinear with the other adjustment variables used. Thus, we adjusted for variables likely to have a direct effect on the faecal contamination of drinking-water (e.g. water source and improved sanitation) rather than rurality and region. Population density may also be a confounding factor (e.g. where increased population density is associated with both increased livestock density and increased levels of drinking-water contamination), although it was not possible to control for this using the available data. Finally, the season of data collection may also influence the results. The majority of survey activities in Bangladesh and Nepal were undertaken during the dry season, when contamination tends to be lower. The Ghana survey activities were carried out over a full calendar year in each region, thus they are representative of conditions across all seasons, but some residual variation in contamination patterns may relate to seasonality (Kostyla et al., 2015).

There may be future opportunities to build on this research, based on further household surveys with linked water quality assessments, or the incorporation of additional questions regarding livestock ownership and presence in water quality surveys. Alternatively, sanitary risk inspection checklists include items regarding access of animals to water sources (e.g. due to inadequate fencing) and the presence of animal excreta within close proximity to the water source (World Health Organization, 2012). Minor modifications to these checklists could enable further analysis of the correlation between livestock presence and faecal contamination in water derived directly from source. However, as far as we are aware no such studies have been conducted as yet.

**Table 6:** Unadjusted and adjusted RRRs related to ownership (versus non-ownership) of large livestock, small livestock and poultry, and categorised numbers of animals owned within each group, for medium and high contamination categories in comparison to the no contamination category in Nepal. N = 1492 (unadjusted) and N = 1448 (adjusted).

Livestock type	Ownership status/number owned	1–31 CFU		>31 CFU		1–31 CFU		>31 CFU	
		Unadjusted RRR (95% CI)	p-value	Unadjusted RRR (95% CI)	p-value	Adjusted RRR^a^ (95% CI)	p-value	Adjusted RRR^a^ (95% CI)	p-value
Large	Non-owner	Ref		Ref		Ref		Ref	
	Owner	1.9 (1.3–2.7)	0.001*	2.7 (1.9–4.0)	<0.001*	1.0 (0.7–1.6)	0.95	1.3 (0.8–2.0)	0.31
Small	Non-owner	Ref		Ref		Ref		Ref	
	Owner	1.6 (1.1–2.3)	0.01*	2.0 (1.3–2.9)	0.001*	0.9 (0.6–1.4)	0.57	0.9 (0.5–1.4)	0.53
Poultry	Non-owner	Ref		Ref		Ref		Ref	
	Owner	1.2 (0.8–1.8)	0.50	1.7 (1.1–2.7)	0.01*	0.7 (0.4–1.1)	0.12	0.8 (0.5–1.4)	0.46
Large	0	Ref		Ref		Ref		Ref	
	1–4	1.9 (1.3–2.7)	0.001*	2.7 (1.8–3.9)	<0.001*	1.0 (0.7–1.6)	0.87	1.2 (0.8–1.9)	0.32
	5+	1.8 (0.9–3.6)	0.11	3.3 (1.6–6.8)	0.002*	0.90 (0.4–2.0)	0.78	1.3 (0.6–3.1)	0.53
Small	0	Ref		Ref		Ref		Ref	
	1–4	1.7 (1.2–2.6)	0.007*	1.8 (1.2–2.8)	0.006*	1.0 (0.6–1.6)	0.97	0.8 (0.5–1.3)	0.43
	5+	1.3 (0.7–2.3)	0.35	2.4 (1.4–4.0)	0.002*	0.6 (0.3–1.3)	0.19	0.9 (0.5–1.8)	0.85
Poultry	0	Ref		Ref		Ref		Ref	
	1–7	1.3 (0.8–2.1)	0.33	1.8 (1.1–2.9)	0.01*	0.7 (0.4–1.3)	0.26	0.8 (0.5–1.4)	0.47
	8+	1.0 (0.5–1.8)	0.95	1.7 (0.9–3.0)	0.10	0.6 (0.3–1.2)	0.14	0.8 (0.4–1.7)	0.64

Ref = reference category. *Statistically significant (p ≤ 0.05). ^a^Adjusted for water source, presence of improved sanitation, water storage status and wealth quintile, but not other livestock ownership categories.

## 5 Conclusions

We have demonstrated significant positive associations between the number of large livestock owned by a household and the risk of faecal contamination of drinking-water at the point of consumption in Ghana and Bangladesh, and between the number of poultry owned and the risk of faecal contamination in Bangladesh, based on the results of nationally representative household surveys. We did not detect significant associations between livestock ownership variables and drinking-water contamination in Nepal. This adds to previous evidence of water contamination by livestock and an increased risk of diarrhoeal disease in individuals who have frequent contact with livestock, although there are still substantial gaps in our understanding. Current prioritisation of water, sanitation and hygiene interventions to reduce consumption of faecally contaminated water and prevent diarrhoeal diseases should also take into consideration the influence of livestock excreta.

## Competing financial interests

The authors declare no competing interests.

## References

[bib0005] Bain R., Cronk R., Wright J., Yang H., Slaymaker T., Bartram J. (2014). Fecal contamination of drinking-water in low- and middle-income countries: a systematic review and meta-analysis. PLoS Med..

[bib0010] Clasen T., Pruss-Ustun A., Mathers C.D., Cumming O., Cairncross S., Colford J.M. (2014). Estimating the impact of unsafe water, sanitation and hygiene on the global burden of disease: evolving and alternative methods. Trop. Med. Int. Health.

[bib0015] Coles C.L., Levy A., Dagan R., Deckelbaum R.J., Fraser D. (2009). Risk factors for the initial symptomatic giardia infection in a cohort of young Arab-Bedouin children. Ann. Trop. Paediatr..

[bib0020] Conan A., O’Reilly C.E., Ogola E., Ochieng J.B., Blackstock A.J., Omore R., Ochieng L., Moke F., Parsons M.B., Xiao L., Roellig D., Farag T.H., Nataro J.P., Kotloff K.L., Levine M.M., Mintz E.D., Breiman R.F., Cleaveland S., Knobel D.L. (2017). Animal-related factors associated with moderate-to-severe diarrhea in children younger than five years in western Kenya: a matched case-control study. PLoS Negl. Trop. Dis..

[bib0025] Cotruvo J.A., Dufour A., Rees G., Bartram J., Carr R., Cliver D.O., Craun G.F., Fayer R., Gannon V.P.J. (2004). Waterborne Zoonoses: Identification, Causes and Control, Emerging Issues in Water and Infectious Disease Series.

[bib0030] Daniels M.E., Shrivastava A., Smith W.A., Sahu P., Odagiri M., Misra P.R., Panigrahi P., Suar M., Clasen T., Jenkins M.W. (2015). Cryptosporidium and giardia in humans, domestic animals, and village water sources in rural India. Am. J. Trop. Med. Hyg..

[bib0035] Dufour A., Bartram J., Bos R., Gannon V.P.J. (2012). Animal Waste, Water Quality and Human Health, Emerging Issues in Water and Infectious Disease Series.

[bib0040] El-Tras W.F., Holt H.R., Tayel A.A., El-Kady N.N. (2015). Campylobacter infections in children exposed to infected backyard poultry in Egypt. Epidemiol. Infect..

[bib0045] Ercumen A., Pickering A.J., Kwong L.H., Arnold B.F., Parvez S.M., Alam M., Sen D., Islam S., Kullmann C., Chase C., Ahmed R., Unicomb L., Luby S.P., Colford J.M. (2017). Animal feces contribute to domestic fecal contamination: evidence from *E. coli* measured in water, hands, food, flies, and soil in Bangladesh. Environ. Sci. Technol..

[bib0050] Georges-Courbot M.C., Cassel-Beraud A.M., Gouandjika I., Monges J., Georges A.J. (1990). A cohort study of enteric campylobacter infection in children from birth to two years in Bangui (Central African Republic). Trans. R. Soc. Trop. Med. Hyg..

[bib0055] Grados O., Bravo N., Black R.E., Butzler J.P. (1988). Paediatric campylobacter diarrhoea from household exposure to live chickens in Lima, Peru. Bull. World Health Organiz..

[bib0060] Kaur M., Graham J.P., Eisenberg J.N.S. (2017). Livestock ownership among rural households and child morbidity and mortality: an analysis of demographic health survey data from 30 Sub-Saharan african countries (2005–2015). Am. J. Trop. Med. Hyg..

[bib0065] Kostyla C., Bain R., Cronk R., Bartram J. (2015). Seasonal variation of fecal contamination in drinking water sources in developing countries: a systematic review. Sci. Total Environ..

[bib0070] Lengerh A., Moges F., Unakal C., Anagaw B. (2013). Prevalence, associated risk factors and antimicrobial susceptibility pattern of Campylobacter species among under five diarrheic children at Gondar University Hospital, Northwest Ethiopia. BMC Pediatr..

[bib0075] Lozano R., Naghavi M., Foreman K., Lim S., Shibuya K. (2012). Global and regional mortality from 235 causes of death for 20 age groups in 1990 and 2010: a systematic analysis for the Global Burden of Disease Study 2010. Lancet.

[bib0080] Mackay E.S.M., Oxford A.E. (1954). Some facultatively anaerobic gram-negative rods from the rumen of the calf and the sheep. J. Gen. Microbiol..

[bib0085] Mahmud M.A., Chappell C., Hossain M.M., Habib M., Dupont H.L. (1995). Risk factors for development of first symptomatic Giardia infection among infants of a birth cohort in rural Egypt. Am. J. Trop. Med. Hyg..

[bib0090] Meays C.L., Broersma K., Nordin R., Mazumder A. (2004). Source tracking fecal bacteria in water: a critical review of current methods. J. Environ. Manage..

[bib0095] Ministry of Food and Agriculture (2010). Agriculture in Ghana Facts and Figures (2009).

[bib0100] Molyneux D., Hallaj Z., Keusch G.T., McManus D.P., Ngowi H., Cleaveland S., Ramos-Jimenez P., Gotuzzo E., Kar K., Sanchez A., Garba A., Carabin H., Bassili A., Chaignat C.L., Meslin F.-X., Abushama H.M., Willingham A.L., Kioy D. (2011). Zoonoses and marginalised infectious diseases of poverty: where do we stand?. Parasites Vectors.

[bib0105] Mosites E.M., Rabinowitz P.M., Thumbi S.M., Montgomery J.M., Palmer G.H., May S., Rowhani-Rahbar A., Neuhouser M.L., Walson J.L. (2015). The relationship between livestock ownership and child stunting in three countries in eastern africa using national survey data. PLoS One.

[bib0110] Prüss-Ustün A., Bartram J., Clasen T., Colford J.M., Cumming O., Curtis V., Bonjour S., Dangour A.D., De France J., Fewtrell L., Freeman M.C., Gordon B., Hunter P.R., Johnston R.B., Mathers C., Mäusezahl D., Medlicott K., Neira M., Stocks M., Wolf J., Cairncross S. (2014). Burden of disease from inadequate water, sanitation and hygiene in low- and middle-income settings: a retrospective analysis of data from 145 countries. Trop. Med. Int. Health.

[bib0115] Randolph T.F., Schelling E., Grace D., Nicholson C.F., Leroy J.L., Cole D.C., Demment M.W., Omore A., Zinsstag J., Ruel M. (2007). Role of livestock in human nutrition and health for poverty reduction in developing countries. J. Anim. Sci..

[bib0120] Schriewer A., Odagiri M., Wuertz S., Misra P.R., Panigrahi P., Clasen T., Jenkins M.W. (2015). Human and animal fecal contamination of community water sources, stored drinking water and hands in rural India measured with validated microbial source tracking assays. Am. J. Trop. Med. Hyg..

[bib0125] Shields K.F., Bain R.E., Cronk R., Wright J.A., Bartram J. (2015). Association of supply type with fecal contamination of source water and household stored drinking water in developing countries: a bivariate meta-analysis. Environ. Health Perspect..

[bib0130] Sustainable Development Solutions Network (2015). Indicators and a Monitoring Framework for Sustainable Development Goals: Launching a Data Revolution for the SDGs.

[bib0135] United Nations General Assembly (2015). Transforming Our World: the 2030 Agenda for Sustainable Development. A/70/L.1.

[bib0140] Wegayehu T., Adamu H., Petros B. (2013). Prevalence of Giardia duodenalis and Cryptosporidium species infections among children and cattle in North Shewa Zone, Ethiopia. BMC Infect. Dis..

[bib0145] World Health Organization, UNICEF (2017). Progress on Drinking Water, Sanitation and Hygiene: 2017 Update and SDG Baselines.

[bib0150] World Health Organization (2012). Rapid Assessment of Drinking-water Quality. A Handbook for Implementation.

[bib0155] World Health Organization (2015). Global Reference List of 100 Core Health Indicators, 2015.

[bib0160] World Health Organization, (1997). Guidelines for Drinking-water Quality, In: Surveillance and Control of Community Supplies.

[bib0165] Wright J., Gundry S., Conroy R. (2004). Household drinking water in developing countries: a systematic review of microbiological contamination between source and point-of-use. Trop. Med. Int. Health.

[bib0170] Wright J., Dzodzomenyo M., Wardrop N.A., Johnston R., Hill A.G., Aryeetey G., Adanu R.M. (2016). Effects of sachet water consumption on exposure to microbially contaminated drinking-water: household survey evidence from Ghana. Int. J. Environ. Res. Public Health.

[bib0175] Yang H., Bain R., Bartram J., Gundry S., Pedley S., Wright J. (2013). Water safety and inequality in access to drinking-water between rich and poor households. Environ. Sci. Technol..

[bib0180] Zambrano L.D., Levy K., Menezes N.P., Freeman M.C. (2014). Human diarrhea infections associated with domestic animal husbandry: a systematic review and meta-analysis. Trans. R. Soc. Trop. Med. Hyg..

